# SARS-CoV-2 Omicron BA.4/BA.5 Mutations in Spike Leading to T Cell Escape in Recently Vaccinated Individuals

**DOI:** 10.3390/v15010101

**Published:** 2022-12-29

**Authors:** Maarten E. Emmelot, Martijn Vos, Mardi C. Boer, Nynke Y. Rots, Cécile A. C. M. van Els, Patricia Kaaijk

**Affiliations:** 1 Centre for Infectious Disease Control, National Institute for Public Health and the Environment (RIVM), 3721 MA Bilthoven, The Netherlands; 2 Faculty of Veterinary Medicine, Utrecht University, 3584 CL Utrecht, The Netherlands

**Keywords:** SARS-CoV-2, Omicron BA.4/BA.5 variants, Omicron BA.1 variant, mutations, T cell response, vaccination, cross-reactivity, immune escape, CD4^+^ T cell epitopes, HLA motif prediction

## Abstract

SARS-CoV-2 Omicron (B.1.1.529) lineages rapidly became dominant in various countries reflecting its enhanced transmissibility and ability to escape neutralizing antibodies. Although T cells induced by ancestral SARS-CoV-2-based vaccines also recognize Omicron variants, we showed in our previous study that there was a marked loss of T cell cross-reactivity to spike epitopes harboring Omicron BA.1 mutations. The emerging BA.4/BA.5 subvariants carry other spike mutations than the BA.1 variant. The present study aims to investigate the impact of BA.4/BA.5 spike mutations on T cell cross-reactivity at the epitope level. Here, we focused on universal T-helper epitopes predicted to be presented by multiple common HLA class II molecules for broad population coverage. Fifteen universal T-helper epitopes of ancestral spike, which contain mutations in the Omicron BA.4/BA.5 variants, were identified utilizing a bioinformatic tool. T cells isolated from 10 subjects, who were recently vaccinated with mRNA-based BNT162b2, were tested for functional cross-reactivity between epitopes of ancestral SARS-CoV-2 spike and the Omicron BA.4/BA.5 spike counterparts. Reduced T cell cross-reactivity in one or more vaccinees was observed against 87% of the tested 15 non-conserved CD4^+^ T cell epitopes. These results should be considered for vaccine boosting strategies to protect against Omicron BA.4/BA.5 and future SARS-CoV-2 variants.

## 1. Introduction

The SARS-CoV-2 Omicron (B.1.1.529) BA.1 variant that emerged in November 2021 resulted in a worldwide surge of infections reflecting its high transmissibility and immune escape potential caused by the multiple mutations in its spike protein [1,2,3,4,5,6,7]. Mid-December 2021-early January 2022, two new lineages, i.e., the Omicron BA.4 and BA.5 subvariants, were identified in South Africa [8]. In Europe the BA.4 and BA.5 Omicron variants were first detected in March 2022. The spike proteins of the BA.4 and BA.5 variants are identical and, although closely related to BA.2, contain additional mutations in the receptor-binding domain [8,9]. The Omicron BA.4/BA.5 subvariants rapidly became the dominant strain in various countries, which indicates their transmission and immune escape advantages compared to other Omicron variants.

Various studies have shown that BA.4/BA.5 virus variants largely evaded neutralizing antibodies induced after immunization with the vaccines based on the ancestral SARS-CoV-2 strain [9,10,11,12,13,14,15,16]. Although mRNA vaccine boosters have shown to be effective in enhancing serum neutralizing activity against BA.4/BA/5 Omicron sublineages [9,10,13,14,15,16], two independent studies showed that neutralizing antibody titers were 15–21 times lower against the BA.4/BA.5 variants as compared to the ancestral SARS-CoV-2 strain at 2–4 weeks after the third dose of the BNT162b2 mRNA vaccine [13,16]. Moreover, neutralization titers against BA.4/BA.5 variants were 3-fold lower compared to neutralization titers against the BA.1 and BA.2 subvariants after this mRNA vaccine booster [9,10,13].

The T cell reactivity against the Omicron variants has shown to be better preserved than neutralizing antibody activity. A modest 10–30% reduction in the T cell response to the spike protein of the Omicron BA.1 subvariant has been reported compared to ancestral spike [17,18,19,20,21]. In our recent study, we showed that while overall T cell responses to Omicron BA.1 were indeed relatively well preserved in vaccinees and convalescent subjects, there was a significant loss of T cell cross-reactivity against specific spike epitopes that carry Omicron BA.1 mutations [22]. At present not much is known about the potential of the more recently emerging Omicron BA.4/BA.5 subvariants to evade memory T cell immunity induced after vaccination. SARS-CoV-2 spike-specific CD4^+^ T cell responses have shown to be more dominant than CD8^+^ T cell responses [23,24,25,26]. Considering all CD4^+^ T cell epitopes of the spike protein, the percentage of conserved epitopes of the BA.4/BA.5 subvariants was quantified to be 74%, and thus based on these calculations a quarter of the CD4^+^ T cell epitopes of spike were non-conserved and contain a BA.4/BA.5 mutation [27]. These mutations in T cell epitopes may lead to a diminished T cell responsiveness against the BA.4/BA.5 subvariants in previously vaccinated individuals.

We analyzed the functional impact of Omicron BA.4/BA.5 spike mutations on the T cell responsiveness to non-conserved epitopes in vaccinees who recently received two original mRNA (BNT162b2) vaccine doses. Results of this study may provide insights useful for the choice between the different newly adapted bivalent vaccines. These bivalent vaccines contain, in addition to ancestral SARS-CoV-2 spike, the BA.1 or BA.4/BA.5 spike variant sequences that better match with the current circulating Omicron variants.

## 2. Materials and Methods

### 2.1. Clinical Samples

Blood samples used were collected from a SARS-CoV-2 vaccination cohort study performed in The Netherlands [22]. Blood samples were taken before Omicron variants were emerging (before October 2021). The study was conducted following the principles of the Declaration of Helsinki, and ethical approval was obtained from the Medical-Ethical Review Committee (MERC) of University Medical Center Utrecht; EudraCT number: 2021-001357-31. Prior to the start of any study-specific procedures, written informed consent was obtained from each of the subjects.

Heparinized blood samples from ten healthy adult participants were used for the present study (5 females/5 males; average age of 29 years (range 23–39 years)). These are the same vaccinated subjects (*n* = 10) that participated in our previous study [22], although new T cell lines were obtained from the peripheral blood mononuclear cell (PBMC) fractions of the blood samples. All study subjects received two doses of the mRNA vaccine based on the ancestral D614G variant spike sequence (BNT162b2), with an interval of 35 days ± 2 days. Blood samples were taken at 4 weeks after the second vaccination. None of the subjects were previously infected with SARS-CoV-2, as confirmed by the absence of pre-vaccination SARS-CoV-2 antibodies [22].

Peripheral blood mononuclear cell (PBMC) fractions were isolated from subjects’ blood samples using Lymphoprep (Progen, Heidelberg, Germany) and cryopreserved at −135 °C.

### 2.2. In Silico Immunogenicity Prediction of CD4+ T Cell Epitope Candidates

In silico immunogenicity prediction based on the ancestral D614G SARS-CoV-2 sequence (UniProtKB: P0DTC2, hereafter, “wildtype-(WT) spike”)) was performed to select universal helper CD4+ T cell epitope candidates of spike. Based on the reported spike mutations of interest for the Omicron BA.4/BA.5 lineages [28,29], the following amino acid mutations and deletions were considered: T19I, Δ24–26, A27S, Δ69–70, G142D, V213G, G339D, S371F, S373P, S375F, T376A, D405N, R408S, K417N, N440K, L452R, S477N, T478K, E484A, F486V, Q493R*, Q498R, N501Y, Y505H, D614G, H655Y, N679K, P681H, N764K, D796Y, Q954H, and N969K. Notably, according to latest WHO information [30] (accessed on 14 October 2022) mutation Q493R* is present in the Omicron BA.1 and BA.2 lineages, but not in the BA.4/BA.5 lineages. For HLA class II motif prediction 19 common HLA class II types were selected, similar as in our previous study [22]. This selection was based on reported HLA class II allelic variants most commonly expressed in the general population [31].

NetMHCIIpan-4.0 (last accessed: 1 October 2022) [32] was used to predict HLA class II binding affinity as well as being an presented HLA class II. Similar approach was used as previously described [22].

For this study, best predicted HLA class II-restricted peptides of WT spike were selected, in which the Omicron BA.4/BA.5 virus variants showed mutations (total of 15 peptides of WT spike) as well as their Omicron BA.4/BA.5 counterparts (all 15-mers; Table 1).

### 2.3. Peptide Synthesis and Peptide Pools Preparation

The 15 selected CD4^+^ T cell epitope candidates of WT spike and the corresponding Omicron BA.4/BA.5 counterparts were synthesized (JPT, Berlin, Germany) (Table 1). In addition, customized peptide pools were generated, one consisting of the 15 selected WT spike T-helper epitope candidates (“WT CD4^+^ pool”), and one peptide pool for the 15 Omicron BA.4/BA.5 T-helper epitope counterparts (“Omicron BA.4/BA.5 CD4^+^ pool”). Peptides were solved in dimethyl sulfoxide (DMSO), and the individual and pooled peptides were diluted with (0.5–1.0 mM and 33.3 µM per peptide, respectively) as previously described in more detail [22].

### 2.4. Preparation of T Cell Lines

T cell lines were generated from the PBMC fractions of all vaccinees as described previously [22]. For this purpose, PBMCs were cultured for 14 days in the presence of “WT CD4^+^ pool” and IL-2. T cell lines were used for functional testing in ELISPOT or flow cytometry.

### 2.5. T Cell Analysis by IFN-ɣ ELISPOT

IFN-ɣ ELISPOT was performed as previously described in detail [22]. T cell lines, plated at a concentration of 5 × 10^4^ cells/well, were restimulated with “WT CD4^+^ pool” or “Omicron BA.4/BA.5 CD4^+^ pool” (1 μM/peptide), or with each of the individual peptides (1 μM). As negative and positive controls, DMSO (0.15%) and PHA (1 µg/mL; Sigma-Aldrich, Darmstadt, Germany) were used, respectively. The number of spots from the negative control (average of triplicate wells) was subtracted from the average spot numbers of triplicate wells of the spike-specific stimulated cells. For the individual peptide testing, for 1 subject, duplicate wells instead of triplicate wells were deployed due to low T cell count.

### 2.6. T Cell Analysis by Flow Cytometry

T cell lines were plated at a concentration of 25 × 10^4^ cells/well in 100 μL AIM-V medium (ThermoFisher, Waltham, MA, USA) with 2% human serum in 96-wells plate, and restimulated with either “WT CD4^+^ pool” or “Omicron BA.4/BA.5 CD4^+^ pool” (1 μM/peptide) for 6 h at 37 °C, 5% CO_2_. Cells were washed and stained as previously described [22] with minor modifications as indicated.

Cells were stained intracellularly for anti-CD154 (clone 24–31; Biolegend, San Diego, CA, USA), and anti-IFN-ɣ (clone 4S.B3; BD Bioscience, Allschwil, Switzerland), anti-IL-2 (clone MQ1-17H12; Biolegend), anti-TNF-α (clone Mab11; Biolegend, San Diego, CA, USA), anti-IL-5 (clone TRFK5) and anti-IL-13 (clone JES10-5A2; Both BD Biosciences, Allschwil, Switzerland). Approximately 133,000 events were acquired on a FACS Symphony A3 analyzer (BD Biosciences, Allschwil, Switzerland). FlowJo (version 10, Tree Star, Ashland, OR, USA) was used for flow cytometry data analysis.

### 2.7. Statistical Analysis

The Wilcoxon signed-rank test was used to compare the paired functional data obtained from T cell lines after in vitro recall with WT spike peptides versus the Omicron BA.4/BA.5 couterparts. Statistical analyses were performed in GraphPad Prism (version 9.3.1; GraphPad Software, San Diego, CA, USA). *p* values less than 0.05 were considered statistically significant.

## 3. Results

### 3.1. Selection of Universal Helper T Cell Epitopes of Spike Containing Omicron BA.4/BA.5 Mutations by In Silico Immunogenicity Prediction

**Figure 1:**
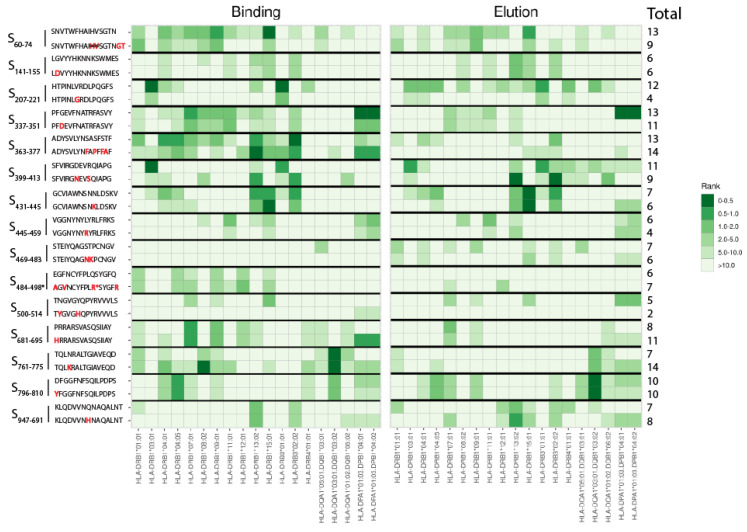
Heatmap presenting binding and elution prediction scores of the 15 selected spike epitope candidates for various common HLA class II alleles. CD4^+^ T cell immunogenicity rank scores of the selected 15-mer peptide sequences of the spike protein of the D614G wildtype (WT) SARS-CoV-2 strain are presented above the rank scores of the corresponding Omicron BA.4/BA.5 variants peptides having a single or more mutations or deletions. Peptide pairs are indicated as location of first and last amino acid position within WT spike protein (S). Amino acid mutations of the Omicron BA.4/BA.5 peptide sequences are presented in red font. The differences in predicted binding affinity rank scores (left panel) or elution scores (right panel) to the various HLA class II alleles are indicated by a color scale. The dark green (i.e., low rank scores) are representative for strongly predicted T cell epitopes for that particular HLA class II allele. On the right, the total number of HLA class II alleles is listed for each peptide with a % rank score <10.0 for either binding affinity prediction or elution. *Q493R: See comment on Q493R mutation in Materials and Methods, Section 2.2 Prediction of CD4^+^ T cell epitope candidates.

When the Omicron (B.1.1.529) BA.5 subvariant became the dominant strain in Europe (June 2022), we started an in silico immunogenicity prediction to select universal helper CD4^+^ T cell epitope candidates of spike based on the ancestral D614G SARS-CoV-2 sequence (wildtype (WT) spike). We only selected T cell epitope regions in which the Omicron BA.4/BA.5 variants contain mutations in spike. The spike proteins of BA.4 and BA.5 are identical. Using the bioinformatic tool NetMHCIIpan-4.0 [32], 15 potential universal T-helper cell epitopes (15-mers) of WT spike were selected based on best prediction scores for 19 common HLA class II alleles (Figure 1 Table 1). According to the IEDB database, the 15 selected candidate epitopes were already identified as proven immunogenic epitope sequences of WT spike [33] (Table 1, two right-hand columns). Notably, fourteen of the fifteen potentially universal, mutated, spike-specific T helper epitope sequences for the BA.4/BA.5 variants were also selected earlier for the BA.1 spike variant [22], either having identical (*n* = 8) or dissimilar (*n* = 6) mutation(s). The prediction and selection pipeline yielded one unique BA.4/BA.5 variants sequence (S_399–413_) not mutated in the BA.1 spike sequence.

Next, predicted immunogenicity scores for the different HLA class II alleles were calculated for the corresponding Omicron BA.4/BA.5 counterparts in order to investigate the impact of the Omicron BA.4/BA.5 mutations on predicted HLA class II binding and likelihood to be naturally presented (based on elution data). Notably, the Omicron BA.4/5 counterparts showed fairly good in silico immunogenicity scores overall for multiple HLA class II alleles (Figure 1). Synthetic standards representing the fifteen WT spike peptide sequences as well as their Omicron BA.4/BA.5 peptide sequences were used in pools or as single peptides in the functional T cell assays of our study.

### 3.2. Functional Impact of Various Omicron BA.4/BA.5 Mutations on T Cell Response to Spike in Prior Vaccinated Subjects

**Figure 2:**
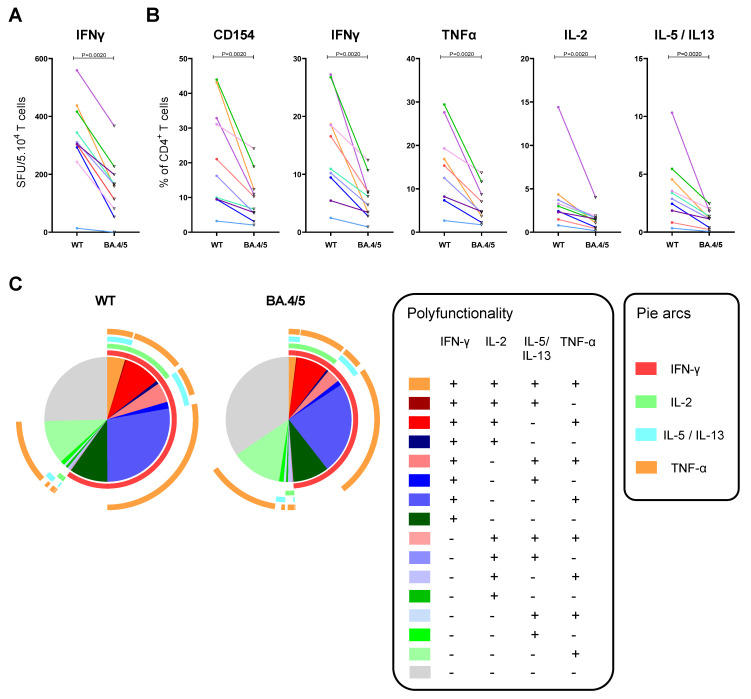
Functional impact of various Omicron BA.4/BA.5 mutations on cross-reactivity to spike of the T cell lines obtained from vacinees. Polyclonal spike-enriched T cell lines were restimulated with “WT CD4+ pool”(WT) or “Omicron BA.4/BA.5 CD4^+^ pool” (BA.4/5), and cultures were analyzed for (**A**) IFN-γ producing spot-forming cells (SFU) per 5 × 10^4^ T cells as quantified by an IFN-ɣ ELISPOT assay (after 24 h restimulation); and for (**B**) percentage of CD154 marker expression, and intracellular IFN-ɣ, TNF-α, IL-2 and IL-5/IL-13 expression of CD4^+^ T cells, as indicated, by flow cytometry (after 6 h restimulation). Responses of each subject are presented by pairs of symbols (closed colored dots for WT restimulation versus open triangles with black border for BA.4/5 restimulation) connected with a colored line per subject. *p* values less than 0.05 were considered statistically significant. (**C**) Pie charts show the proportion of activated CD145^+^/CD4^+^ T cells (median values) secreting no, one, or multiple cytokines upon WT or BA.4/5 stimulation, as color-indicated.

To be able to interrogate the T cells reactive to the fifteen selected WT spike epitopes and test their cross-reactive potential to the corresponding mutated Omicron BA.4/BA.5 peptide sequences, antigen-specific T cell enrichment was employed by in vitro stimulation with the “WT CD4^+^ pool”. The spike-specific IFN-ɣ-producing cells in the expanded T cell lines, that were obtained from PBMCs of 10 vaccinated subjects also selected for our previous study [22], were enumerated by an ELISPOT. A significant decrease in of SARS-CoV-2 spike-specific IFN-ɣ-producing cell frequencies was found after in vitro peptide recall with the “Omicron CD4^+^ pool” compared to the “WT CD4^+^ pool” (1.9-fold decrease; respectively, median of 162 versus 308 SFU/5 × 10^4^ T cells; *p* = 0.0020) (Figure 2A).

In parallel, the “WT CD4+ pool” expanded T cell lines were tested for CD4^+^ and CD8^+^ T cell responses after in vitro peptide restimulation using a flow cytometry-based T cell assay with combined activation-induced marker (AIM) and intracellular cytokine staining (ICS) (Figure 2B). High percentages of SARS-CoV-2-specific CD4^+^/CD154^+^ activated T cells were measured after 6 h restimulation with the “WT CD4^+^ pool” (median of 19% of total CD4^+^ population), that were significantly lower after restimulation with “Omicron BA.4/BA.5 CD4^+^ pool” (8.5%; *p* = 0.0020). In addition, in line with the IFN-ɣ ELISPOT results, all 10 vaccinees showed significantly lower percentages of IFN-ɣ^+^ CD4^+^ T cells after stimulation with “Omicron BA.4/BA.5 CD4^+^ pool” compared to “WT CD4^+^ pool” (2.5-fold decrease; respectively, 5.5% versus 14% of total CD4^+^ T cells; *p* = 0.0020). Similar patterns were observed for intracellular TNF-α expression of the CD4^+^ T cell population (2.9-fold decrease; 4.8% vs. 14%, for, respectively, “Omicron BA.4/BA.5 CD4^+^ pool” and “WT CD4^+^ pool” stimulation; *p* = 0.0020). Additionally, for the other tested cytokines, IL-2, and IL-5/IL-13, a significantly lower intracellular expression was found in T cells of vaccinees after stimulation with “Omicron BA.4/BA.5 CD4^+^ pool” compared to “WT CD4^+^ pool”, although the percentages of cytokine-positive CD4^+^ T cells were considerably lower than the percentages of IFN-ɣ- and TNF-α-positive CD4^+^ T cells (Figure 2B).

In the population of CD4^+^/CD154^+^ activated T cells, a higher proportion of cells did not express any of the cytokines after stimulation with “Omicron BA.4/BA.5 CD4^+^ pool” compared to “WT CD4^+^ pool” (BA.4/BA.5: 34% versus WT: 25%) (grey part in pie chart, Figure 2C). However, in general, spike-specific activated T cells showed a similar pattern of polyfunctionality against the BA.4/BA.5 peptide sequences (Figure 2C). Some of the activated CD4^+^ T cells produced each of the tested cytokines (i.e., IFN-ɣ, TNF-α, IL-2, and IL-5/IL-13) upon stimulation with the “CD4^+^ pools”, indicating that both Th1 and Th2 cytokines could be induced by the universal helper epitopes of spike protein.

As expected, recall responses by CD4^+^ T cells and hardly by CD8^+^ T cells were observed after stimulation with the universal helper epitopes. The median percentages of the CD3^+^/IFN-ɣ^+^ T cells after “WT CD4^+^ pool” stimulation were 90% for CD4^+^ and 2% for CD8^+^ T cells; remaining IFN-ɣ^+^ cells were mostly of CD3^+^/CD4^-^/CD8^-^ phenotype.

Taken together, these results indicate that the spike-enriched T cell lines from recently vaccinated subjects were highly activated, and showed abundant (simultaneous) expression of IFN-ɣ, TNF-α, IL-2 and/or IL-5/IL-13, upon stimulation with the pool of the 15 universal helper epitopes of WT spike. In contrast, T cell reactivity, mainly present in the CD4^+^ population, was significantly reduced against the corresponding pool of peptides with Omicron BA.4/BA.5 mutations.

### 3.3. Omicron BA.4/BA.5 Mutations in Individual Spike Epitopes Lead to Reduced Cross-Reactivity

**Figure 3:**
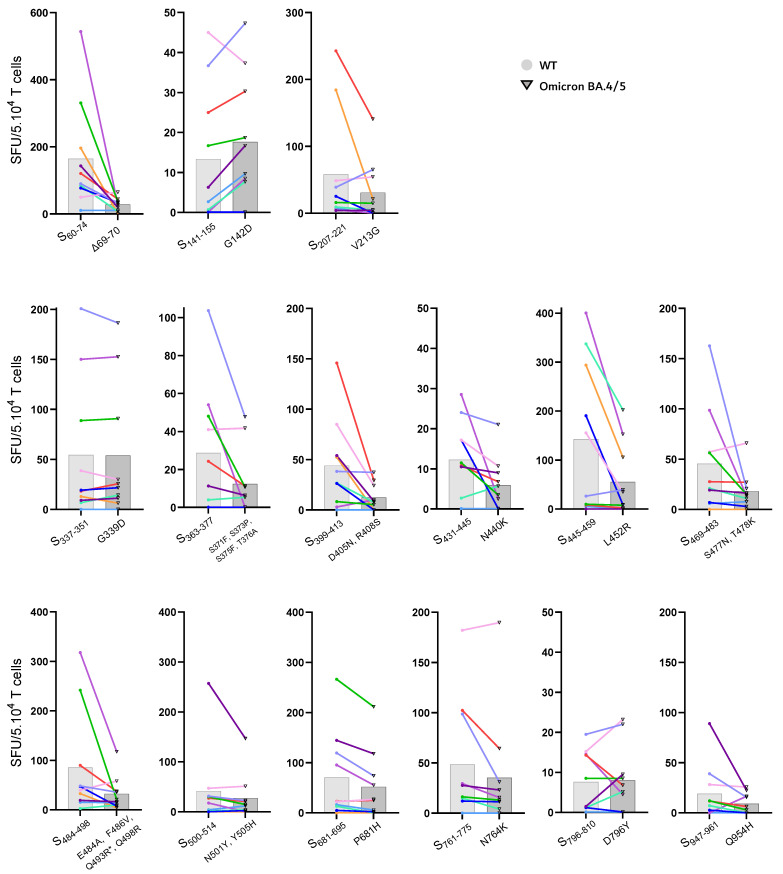
In T cell lines obtained from vaccinees, IFN-ɣ responses are reduced to various, but not all, spike epitopes containing BA.4/BA.5 mutations compared to the original D614G wildtype (WT) spike epitopes. Polyclonal spike enriched T cell lines were restimulated for 24 h with individual WT spike epitopes or corresponding sequences of the Omicron BA.4/BA.5 variants and analyzed for IFN-γ producing spot-forming cells (SFU) per 5 × 10^4^ T cells as quantified by an IFN-ɣ ELISPOT assay. Responses of each subject are represented by pairs of symbols (closed colored dots for WT restimulation versus open triangles with black border for BA.4/5 restimulation) connected with a colored line per subject. On the *X*-axis, WT (left) and Omicron BA.4/BA.5 (right) peptide pairs are indicated as location of first and last amino acid position within WT spike protein (S), and the Omicron BA.4/BA.5 amino acid mutation(s) or deletions. Notice, different *Y*-axis scales were used to be able to optimally visualize differences between subjects for each spike epitope.

Next, an IFN-ɣ ELISPOT was performed to identify which of the individual mutated BA.4/BA.5 spike epitopes were responsible for reduced T cell cross-reactivity (Figure 3). In general, the enriched T cell cultures obtained from the recently vaccinated subjects showed good memory T cell responses to most of the 15 WT spike epitopes, indicating good immunogenicity of the selected universal helper epitopes. As expected, differences in the response per individual peptide were observed among the 10 vaccinees, reflecting variations in the subject’s HLA type and T cell epitope repertoire. Significant reduction in IFN-ɣ^+^ T cell frequencies in the cell lines obtained from vaccinees was observed after stimulation with Omicron BA.4/BA.5 variants peptide sequences compared to WT spike peptides, for the following epitopes: S_60–74_ (responsible deletions: Δ69–70), S_363–377_ (responsible mutations: S371F, S373P, S375F, and/or T376A), S_399–413_ (D405N and/or R408S), S_431–334_ (N440K), S_445–459_ (L452R), S_469–483_ (S477N, and/or T478K), and S_484–498_ (E484A, Q493R (present in BA.1 not BA.4/BA.5), F486V, and/or Q498R). For most of the other epitopes, in only a few subjects, a clear decline in responsiveness to the Omicron BA.4/BA.5 peptides was found. Only for the two (S_141–155_ (with mutation G142D) and S_337–351_ (G339D)) Omicron BA.4/BA.5 peptides, a similar recall response was found as compared to the WT counterparts, indicating a preserved memory T cell response to these two Omicron BA.4/BA.5 spike epitopes (Figure 3, Table 2).

In our previous study we also observed a marked loss of T cell cross-reactivity to spike epitopes harboring Omicron BA.1 mutations [22], a panel of 20 epitopes with considerable overlap with the currently selected 15 BA.4/BA.5 spike epitope sequences (*n* = 14), based on identical (*n* = 8) or dissimilar mutations (*n* = 6). Comparing the impact of the BA.4/BA.5 versus the BA.1 mutations in the spike peptides on the T cell recall response in the two studies based on separately generated T cell lines from vaccinees, allows identification of BA.4/BA.5 and/or BA.1 mutations responsible for the reduced T cell responsiveness (Table 2). In this way, we can unravel which of the mutations in Omicron BA.4/BA.5 and/or BA.1 are responsible for the observed decline in T cell reactivity against the corresponding epitopes with Omicron mutations (listed in right column of Table 2). The preserved T cell response found to the BA.1 epitope of S_445–459_ (G446S) [22] is in contrast with the clearly reduced response found to this BA.4/BA.5 epitope carrying the L452R mutation (Figure 3). The uniform T cell responses found across our two studies against the matching BA.4/BA.5 and BA.1 epitopes (*n* = 8) in the same set of 10 vaccinees [22] confirm the consistency in our results, even as new enriched T cell lines were generated against a slightly different pool of WT spike peptides. Only the S_796–810_ epitope, that induced limited T cell responses in both studies, showed in the present study a low T cell response that slightly declined in a few subjects in response to the BA.4/BA.5 variant epitope, possibly indicating some T cell escape, whereas this was not observed in our previous study [22]. The other identical BA.4/BA.5 and BA.1 epitope sequences showed all similar patterns in T cell responsiveness across the two studies.

Taken these results together various BA.1 and BA.4/BA.5 mutations could be identified that lead to a reduced epitope-specific T cell response, as listed in Table 2.

## 4. Discussion

New sublineages of Omicron (B.1.1.529) with diverse mutations in spike protein are gaining prevalence, suggesting a further increase in virus transmissibility and immune escape. Whereas neutralizing antibodies induced after vaccination with the original vaccine have shown to be largely evaded by BA.4/BA.5 variants [9,10,11,12,13,14,15,16], less evidence is available for T cell escape. T cell immunity is critical for memory responses against SARS-CoV-2 infection to prevent severe disease [26,34,35,36,37]. Thus, establishing the impact of the Omicron BA.4/BA.5 virus variants on T cell cross-reactivity is important to guide vaccination strategies for prevention of disease by these SARS-CoV-2 variants of concern. Recently, we showed that several mutations in spike protein of the Omicron BA.1 variant lead to diminished functional T cell responses to individual spike epitopes in vaccinated subjects [22]. Nevertheless, we also confirmed that, despite >30 mutations in spike of the SARS-CoV-2 Omicron BA.1 variant, the overall CD4^+^ memory T cell response against the whole protein sequence of ancestral spike was relatively well preserved in recently vaccinated persons and in convalescent individuals [22]. This is in agreement with other studies reporting that T cell responses to the Omicron BA.1 spike protein show, on average, a modest decrease of 10–30% compared to the ancestral SARS-CoV-2 spike protein [17,18,19,20,21]. Based on identified CD4^+^ T cell epitopes of spike available in the Immune Epitope Database (IEDB), the percentage of conserved epitopes of the BA.4/BA.5 subvariants was quantified to be 74% [27].

The spike protein of the BA.4/BA.5 variants has 22 mutations in common with BA.1 spike. Even more spike mutations of Omicron BA.4/BA.5 match with those of the BA.2 variant. However, the BA.4/BA.5 spike also possesses a few mutations that are distinctive from previous Omicron sublineages, i.e., L452R and F486V. We identified 15 universal T helper cell epitope sequences in WT spike, based on high and broad HLA class II prediction scores, that contain one or more Omicron BA.4/BA.5 mutations.

We show here that CD4^+^ T cells expanded with the 15 WT spike epitopes, obtained from PBMCs of subjects recently vaccinated with the original WT strain-based mRNA vaccine, do indeed recognize most of the selected epitopes. This confirms the good immunogenicity and broad recognition of the 15 selected spike epitopes. However, in one or more vaccinated subjects, a considerable loss of T cell cross-reactivity was found against 87% of the BA.4/BA.5 spike epitope counterparts. Twenty-one mutations or deletions in Omicron BA.4/BA.5 spike (excluding Q493R, only present in BA.1) could be held responsible for the diminished cross-reactivity, 13 of these mutations/deletions are identical to BA.1 mutations (Table 2). However, also, the BA.4/BA.5 mutations, V213G, D405N and/or R408S, L452R, and possibly S371F, T376A and/or F486V, not present in BA.1, caused a reduction in T cell responsiveness (Table 2).

The spike sequences S_445–459_ and S_446–465_ (including the S_445–459_ epitope sequence), have also been identified by others as an immunodominant T cell epitope [38,39]. The L452R spike mutation (leucine-arginine replacement), found in the BA.4/BA.5 variants and responsible for diminished cross-reactivity, is a spike mutation that was already present in the SARS-CoV-2 variants, i.e., Kappa (B.1.617.1) and Delta (B.1.617.2). In the Omicron BA.2.12.2 variant, the leucine on position 452 of spike protein sequence is replaced by glutamine (L452Q) as had been found for Lambda (C.37) variant as well. This L452Q mutation could possibly also lead to T cell escape. Previously, we showed that the G446S mutation in the BA.1 epitope of S_445–459_ did not affect the T cell response [22].

Consistent with our HLA prediction scores, Sankaranarayanan et al. found that the majority of mutated CD4^+^ T cell spike epitopes retain the HLA restriction pattern of their native epitopes, and claim that this is suggestive for a conserved T cell response [40]. However, our data reveal that a retained favorable HLA restriction pattern is not necessarily associated with maintenance of T cell recognition of the mutated epitope. For instance, the Omicron BA.4/BA.5 variants sequence of S_761–775_ showed reduced T cell reactivity compared to the WT sequence, while the HLA class II binding prediction score of the mutated epitope was even better. This indicates that not only HLA binding, but also an impaired recognition of the HLA–peptide complexes by the T cell receptor (TCR), may contribute a reduced T cell reactivity.

In our study, blood samples were taken 4 weeks after subjects received their last dose of the primary series of the mRNA-based vaccine based on the ancestral WT spike sequence. Most people today have had more than two vaccinations, and often additional (Omicron) infection(s), which will also affect T cell responses and may increase reactivity to spike of the Omicron lineages. Furthermore, in August/September 2022, the first bivalent mRNA vaccines, comprising original and Omicron BA.1 spike mRNAs, were authorized for use as booster dose. These vaccines were developed to enhance immunity to the ancestral WT strain as well as inducing Omicron BA.1 variant-specific immunity. Although the Omicron BA.4/BA.5 spike sequence shares 22 amino acid mutations/deletions with the BA.1 variant, individuals previously infected with the BA.1 variant showed significant escape of antibody neutralizing immunity to BA.4/BA.5 [12,41,42]. This might indicate that BA.1-containing vaccine boosters may not result in broad-spectrum protection to the BA.4/BA.5 Omicron variants either [12]. On the other hand, a study in Qatar showed that prior infection with Omicron (BA.1 or BA.2) was 78% effective at preventing BA.4 and BA.5 reinfection and 76% effective at preventing symptomatic reinfection [43]. Nevertheless, first studies show that the bivalent Omicron BA.1-containing vaccine boosts neutralizing antibody responses against Omicron BA.1 and BA.4/BA.5 to levels that were superior to those boosted with the original vaccine [44]. Interestingly, BA.1 infection also induced new clones of BA.1-specific antibodies that potently neutralize BA.1. However, these neutralizing antibodies were largely evaded by BA.2 and BA.4/BA.5 variants owing to D405N and F486V mutations [12,42]. Shortly after approval of the first bivalent mRNA COVID-19 vaccines, new BA.4/BA.5-containing bivalent vaccines have now also been authorized for emergency use. Whether these Omicron-containing booster vaccines could result in stronger T cell responses to Omicron variants is not yet known.

Perhaps infection or vaccination with Omicron variant strains can also induce T cells reactive against novel epitopes of spike. Our data show that several T-helper epitopes harboring Omicron BA.4/BA.5 mutations were predicted to bind even better to certain HLA class II molecules than the WT spike counterparts (Figure 1). Exposure to these Omicron-specific novel epitopes by vaccination and/or infection could improve T cell immunity to Omicron variants. Nevertheless, it should be monitored whether Omicron-specific B- and T cell immune responses, if induced, do not undermine the responsiveness to more pathogenic variants, like Delta. Several studies have shown that the first encounter with Omicron either through infection or vaccination, but without a previous SARS-CoV-2 infection or vaccination, results in neutralizing responses predominantly directed against Omicron with more limited neutralization against earlier VOCs [45,46,47].

In summary, our study shows that several BA.4/BA.5 mutations in the spike protein lead to a reduced responsiveness of epitope-specific T cells in subjects that received two doses of a mRNA vaccine based on the ancestral WT spike sequence. Other currently circulating Omicron sublineages, such as BA.2.75, BA.4.6, BQ.1.1 and XBB, share many of these spike mutations, making our findings also relevant for the impact of the T cell response on these emerging Omicron variants. Future research should indicate whether Omicron infection(s) or booster dose(s) of the new bivalent Omicron vaccine may induce T cells reactive to novel epitopes of spike that may lead to an enhanced T cell immunity to Omicron.

## Figures and Tables

**Table 1:** List of 15 selected universal helper epitope candidates of WT spike and Omicron BA.4/BA.5 counterparts.

pos	Mutation	WT Sequence	BA.4/BA.5 Sequence	Spike Domain	IEDB	IEDB Pos
S_60–74_	Δ69–70	SNVTWFHAIHVSGTN	SNVTWFHAISGTN**GT** ^2^	S1	1310701	S_61–75_
S_141–155_	G142D	LGVYYHKNNKSWMES	L**D**VYYHKNNKSWMES ^2^	S1	1310575	S_141–155_
S_207–221_	V213G	HTPINLVRDLPQGFS	HTPINL**G**RDLPQGFS ^2^	S1	1309123	S_206–220_
S_337–351_	G339D	PFGEVFNATRFASVY	PF**D**EVFNATRFASVY ^3^	S1/RBD	1310312	S_336–350_
S_363–377_	S371F, S373P S375F, T376A	ADYSVLYNSASFSTF	ADYSVLYN**F**A**P**F**FA**F ^2^	S1/RBD	1330442	S_364–378_
S_399–413_	D405N, R408S	SFVIRGDEVRQIAPG	SFVIRG**N**EV**S**QIAPG	S1/RBD	1330436	S_398–413_
S_431–445_	N440K	GCVIAWNSNNLDSKV	GCVIAWNSN**K**LDSKV ^3^	S1/RBD	1310437	S_431–445_
S_445–459_	L452R	VGGNYNYLYRLFRKS	VGGNYNY**R**YRLFRKS ^2^	S1/RBD	1073698	S_445–459_
S_469–483_	S477N, T478K	STEIYQAGSTPCNGV	STEIYQAG**NK**PCNGV ^3^	S1/RBD	1313689	S_469–483_
S_484–498_^1^	E484A, F486V, Q493R*, Q498R	EGFNCYFPLQSYGFQ	**A**G**V**NCYFPL**R**SYGF**R**^2^	S1/RBD	1397221	S_483–500_
S_500–514_	N501Y, Y505H	TNGVGYQPYRVVVLS	T**Y**GVG**H**QPYRVVVLS ^3^	S1/RBD	1540449	S_496–515_
S_681–695_	P681H	PRRARSVASQSIIAY	**H**RRARSVASQSIIAY ^3^	S1/S2	1394068	S_680–696_
S_761–775_	N764K	TQLNRALTGIAVEQD	TQL**K**RALTGIAVEQD ^3^	S2	1310863	S_761–775_
S_796–810_	D796Y	DFGGFNFSQILPDPS	**Y**FGGFNFSQILPDPS ^3^	S2	1312421	S_797–811_
S_947–961_	Q954H	KLQDVVNQNAQALNT	KLQDVVN**H**NAQALNT ^3^	S2	1310448	S_946–960_

^1^ Q493R: See comment on Q493R mutation in Materials and Methods, Section 2.2 Prediction of CD4+ T cell epitope candidates. ^2^ Omicron BA.4/BA.5 spike sequence with dissimilar mutation(s) to WT spike positions as BA.1 spike (as studied in [22]). ^3^ Omicron BA.4/BA.5 spike sequence with identical mutation(s) to WT spike positions as BA.1 spike (as studied in [22]). The location of the peptides (all 15-mers) are indicated as the position (pos) of the first and last amino acid of spike. Mutations in Omicron BA.4/BA.5 sequences compared to WT spike sequence are shown as bold red font. Identified immune epitope database (IEDB) epitope sequences of SARS-CoV-2 spike protein, shown as IEDB identifier number, that were tested positive in a T cell assay according to information at http://www.iedb.org [33] (accessed: 14 October 2022, input: Epitope source Organism, SARS-CoV-2 (ID: 2967049); Epitope source Antigen, Spike glycoprotein [P0DTC2] (SARS-CoV-2); Host, Human; Assay, T cell; MHC restriction, Class II). The location of the peptide from IEDB (IEDB pos) is shown as first and last amino acid position of the spike protein. Abbreviations: WT, spike of D614G wildtype reference strain; BA.4/BA.5, spike of Omicron BA.4/BA.5 strain; IEDB, The Immune Epitope Database; S1, the S1 subunit of spike protein (S14–684); S2, the S2 subunit of spike protein (S686–1273); RBD, the receptor binding domain of spike protein (S319–541).

**Table 2:** List of BA.1 and BA.4/BA.5 mutations that can be responsible for partial or complete CD4+ T cell escape.

Spike Location	BA.4/5 Mutation	BA.4/5 Escape *	BA.1 Mutation	BA.1 Escape	Mutations Responsible for CD4^+^ T Cell Escape
**S_60–74_**	Δ69–70	Yes	A67V, Δ69–70	Yes	Definitely: Δ69–70 Possibly: A67V
**S_87–101_**		-	T95I	Yes	T95I
**S_141–155_**	G142D	No	G142D, Δ143–145	Yes	Δ143–145
**S_207–221_**	V213G	Yes	Δ211, L212I, 214EPEins	Yes	Δ211, L212I and/or 214EPEins Definitely: V213G
**S_337–351_**	G339D	No	G339D	No	x
**S_363–377_**	S371F, S373P S375F, T376A	Yes	S371L, S373P, S375F	Yes	S371F, S371L, S373P S375F and/or T376A
**S_399–413_**	D405N, R408S	Yes		-	D405N and/or R408S
**S_431–445_**	N440K	Yes	N440K	Yes	N440K
**S_445–459_**	L452R	Yes	G446S	No	L452R
**S_469–483_**	S477N, T478K	Yes	S477N, T478K	Yes	S477N and/or T478K
**S_484–498_**	E484A, F486V, Q493R *, Q498R	Yes	E484A, Q493R, G496S, Q498R	Yes	E484A, F486V, Q493R, G496S, and/or Q498R
**S_492–506_**		-	Q493R, G496S, Q498R, N501Y, Y505H	No	x
**S_500–514_**	N501Y, Y505H	Yes	N501Y, Y505H	Yes	N501Y, Y505H
**S_540–554_**		-	T547K	Yes	T547K
**S_681–695_**	P681H	Yes	P681H	Yes	x
**S_761–775_**	N764K	Yes	N764K	Yes	N764K
**S_796–810_**	D796Y	Yes	D796Y	No	Possibly: D796Y
**S_852–866_**		-	N856K	Yes	N856K
**S_947–961_**	Q954H	Yes	Q954H	Yes	Q954H
**S_967–981_**		-	L981F	Yes	L981F
**S_973–987_**		-	L981F	Yes	L981F

* BA.4/BA.5 or BA.1 T cell escape (based on this study or on our previous study [22], respectively) is described as follows: when T cells of 1 or more subjects respond to the WT spike epitope (≥10 IFN-ɣ SFU per 5 × 10^4^ T cells) and also show a ≥1.5-fold reduction of IFN-ɣ-producing T cells against the corresponding epitope of the Omicron variant. BA.4/BA.5 or BA.1 spike mutations that can be responsible for CD4^+^ T cell escape are presented in green boxes, BA.4/BA.5 or BA.1 mutant sequences that are not involved in CD4^+^ T cell escape are presented in red boxes; BA.4/BA.5 spike mutations that are not present in BA.1 spike are presented in red font, BA.1 spike mutations that are not present in BA.4/BA.5 spike are presented in blue font. Q493R*: See comment on Q493R mutation in Materials and Methods, Section 2.2 Prediction of CD4^+^ T cell epitope candidates.

## Data Availability

Upon request, raw data sets supporting the conclusions of this study will be made available by the authors taking into consideration privacy and ethical rights of the participants.
